# On Psychotherapeia

**Published:** 1854-07

**Authors:** W. C. Dendy


### SELECTIONS.

From the Medical Times and Gazette.

*On Psychotherapeia.*—By W. C. DENDY. Esq.

We quote the commencing passages as the psychological basis of the essay. "When Plato wrote these words, '*Nec totum corpus (curibis) sine anima,*' he recorded a truth which few probably will deny, but the principle of which, in the practice of medicine, has been constantly blinked or set aside. This error has been committed, not only from deficient appreciation of the influence of mind, and especially that one of its faculties which we term volition, but also from a notion that the psychologist speaks and writes of intellect as an abstraction, and not as that intimite blending of mind and matter which has laid the basis of modern psychology, and especially the theory of insanity. What the blood is to a secreting gland, the spirit is to the brain. The gland forms its especial product from the blood; the brain acting with spirit produces mind." While dwelling on the pathological influence of mind on tissue, the author thus traced the course of a simple thought: "The sensations it often induces are those which, if in higher degree, or more permanent, would become symptoms or indications of disorder. What is a chill (as of fear) but a rigor, like that of ague—its cause cardiac congestion?—What a throb, but that exalted innervation, which, if protracted, might produce cardiac hypertrophy? What the flush, but that hyperemic condition which might terminate in inflammation?" The physiological prophylatic, and therapeutical influence of the mens sana was next referred to, and especially the principle of psychical antagonism opposing, for instance, the effects of anxiety fear, melancholy, remorse, &c. The principle of mesmerism, and its prototypes, were entirely psychical, and should not be blinked by medical philosophers, who thus apathetically permit the mercenary impostor to gain the ascendant. Even the organic functions dependent on special and ganglionic influences may be mentally excited. Even peristaltic action, the salivary and spermatic secretions, may be induced by peculiar studies and thoughts. The depressing effects of grief and fear be influential in checking hemorrhage, or in removing acute neuralgia, as in inflammatory toothache, &c , and the effects of shame, by derivation, relieving the condition of internal hypermia, &c. Mr. Dendy referred to the extraordinary effects of terror in the case of the previously speechless son of Crœsus, who on the uplifting of an assassin's arm exclaimed, "Slave, I kill not Crœsus," and to others. Hope is not only felt in the heart but is synchronously the cause of a vigorous circulation. The pulse, respiration, and powers of a soldier during an inglorious retreat were contrasted with those of the victorious pursuers. In the hospital also of a defeated army the healing process is far more slow and imperfect than in that of the conqueror. The very interesting accounts of scorbutics in Lord Anson's voyage, and the pious fraud of the Prince of Orange at the siege of Breda, were apt illustrations of the effect of confidence and hope. To prove the intense effect of joy, Mr Dendy related the case of a young lady, who had been long afflicted with complete hysterical aphonia, whose vocal organs were brought for a moment into play by a promise of one of the costly jewels in the exhibition, if she would pronounce its name. She gained the prize. Mr. Dendy next alluded to that prevalent error of human nature, automania or wrong notions of self. Several interesting cases were adduced from the author's practice, from de Bloismont, &c . as illustrations of the potent effect of diversion or obeyance of the mind as a remedy for maladies which would perfectly resist the materia medica. If the current of morbid innervation be intercepted, the mind would forget the malady, which, according to the theory of Berkely, would then cease to exist. This is a subject really of practical importance. In allusion to other cases of maladie imaginarie, one melancholy instance was mentioned of a medical gentleman under the author's care, in whom, in the intervals of extensive practice, the automania that syphilis was corroding his osseous system, at length terminated in suicide. The notion was perfectly groundless, and *gnothi seauton* would have saved him. Congested lung, when instinct fails in its duty, must be relieved by voluntary effort; we must not forget to breathe.—The consequent collapse of the air-cells will not only increase congestion but even favor the development of tubercle. By the due expansion of cells, the granule or germ in the pulmonary parenchyma may be subdued and kept down. The cases of Bateman and John Hunter might have formed fatal illusions, had not the one been incessantly roused from slumber, and the other set himself to the voluntary inflation of his lungs. On this principle, sleep may be sometimes perilous in pulmonary disorder, as it withdraws volition. Probably this may have been the causa mortis in old asthmatic persons who, having long endured a sort of chronic atalectasis, have been discovered dead in their beds. The paper concludes thus: 'The basis of my remarks in this proposition,—

A mere thought instantly induces a physical change, it may be, even in the crasis of the blood. By directing or averting such thought to or from disordered structure or function, we may constantly avail ourselves of a valuable auxiliary in the practice of our intricate science."

